# Defining Subclinical Acute Kidney Injury Within 72 Hours After Minimally Invasive Esophagectomy in Prone Position and Its Association With Outcomes: An Exploratory Study

**DOI:** 10.7759/cureus.103141

**Published:** 2026-02-07

**Authors:** Seiji Ishikawa, Junko Hirashima, Makiko Hiroyama, Shojiro Ozato, Masayuki Watanabe, Katsuyuki Terajima

**Affiliations:** 1 Department of Anesthesiology and Pain Medicine, Juntendo University, Tokyo, JPN; 2 Department of Anesthesiology, Cancer Institute Hospital of Japanese Foundation for Cancer Research, Tokyo, JPN; 3 Department of Gastroenterological Surgery, Cancer Institute Hospital of Japanese Foundation for Cancer Research, Tokyo, JPN

**Keywords:** acute kidney injury, esophageal cancer, mechanical ventilation, minimally invasive esophagectomy, postoperative complication

## Abstract

Introduction: This study aimed to investigate the effects of slight increases in the serum creatinine concentration (sCr) postoperatively that do not meet the diagnostic criteria for acute kidney injury (AKI) on outcomes in patients undergoing minimally invasive esophagectomy (MIE) in the prone position.

Methods: A retrospective cohort study was carried out on patients who underwent MIE between January 2010 and December 2024. Patients who underwent MIE were categorized into three groups: those diagnosed with AKI based on the Kidney Disease: Improving Global Outcomes creatinine criteria within 72 hours after surgery (AKI group); those with a postoperative percentage increase in serum creatinine (ΔsCr%) of 30% or more compared with baseline (subclinical AKI group); and those with a ΔsCr% <30% (reference group). Patient outcomes were compared among the groups.

Results: Of 933 patients, 33, 12, and 888 patients were classified into the AKI, subclinical AKI, and reference groups, respectively. The proportion of patients with postoperative hospital stay ≥25 days and the proportion of patients who required mechanical ventilation after surgery were significantly higher in the AKI group (51.5%, 27.3%) and the subclinical AKI group (50.0%, 25.0%) than in the reference group (18.6%, 3.3%) (p<0.0167, Bonferroni correction). No statistically significant differences were identified between the AKI and subclinical AKI groups with respect to these outcomes.

Conclusions: The incidence of subclinical AKI, defined based on ΔsCr% after MIE, was 1.3%. Subclinical AKI patients were significantly more likely to require postoperative mechanical ventilation. To prevent worsening outcomes after MIE, not only AKI, but also subclinical AKI should be targeted for prevention.

## Introduction

Postoperative acute kidney injury (AKI) has been recognized as a factor that adversely affects both short-term [1,2] and long-term [3,4] outcomes in a wide range of surgical settings. We have previously shown that postoperative AKI within 72 hours after esophagectomy is associated with poor outcomes, such as increased hospital length of stay and re-intubation [5]. In addition to AKI, the problem of slight increases in serum creatinine concentration (sCr) that do not meet the diagnostic criteria for AKI immediately after major surgery being associated with worsening outcomes has been investigated as subclinical AKI, primarily in cardiac surgery [6,7]. The reason why subclinical AKI is associated with worsening outcomes is unclear, but it has been suggested that hemodilution associated with fluid infusion and cardiopulmonary bypass during cardiac surgery, i.e., a decrease in sCr during the perioperative period, may cause an underestimation of the decrease in renal function.

Minimally invasive esophagectomy (MIE)-encompassing thoracoscopic, laparoscopic, mediastinoscopic, and robot-assisted techniques for esophageal cancer-is gradually gaining widespread adoption across the globe [8]. Although it has been shown that patients who underwent MIE had a lower incidence of complications, such as in-hospital mortality and re-operation, as well as a shorter postoperative hospital stay than patients who underwent open surgery, the duration of anesthesia was shown to be longer [9]. In fact, the median duration of anesthesia exceeded 10 hours, and the median amount of fluid administered exceeded 3.5 L in our previous study of MIE [10]. Therefore, after MIE, sCr may decrease due to dilution of creatinine by intravenous fluids and reduced creatinine production from muscles because food intake is not resumed immediately [11]. Because the diagnosis of AKI is generally based on changes in sCr, there is a possibility that in such circumstances the decrease in renal function may be underestimated. However, there have been very few reports of the effects of subclinical AKI on outcomes after non-cardiac surgery [12], and to the best of our knowledge, there are no reports following MIE.

In our previous study, we investigated the incidence, risk factors, and impact on outcomes of acute kidney injury after esophagectomy, including not only MIE but also thoracotomy [5]. In this study, we narrowed our focus to MIE and investigated subclinical AKI that occurs after surgery. The objectives of the present study were fourfold. First, in an exploratory manner, we sought to identify a definition of subclinical AKI after MIE that is associated with worsening outcomes. Based on the definition derived from this exploratory step, the subsequent objectives were to determine the incidence of subclinical AKI within 72 hours after MIE; to characterize subclinical AKI in relation to patient background and anesthesia and surgery-related factors; and to evaluate the impact of subclinical AKI on postoperative outcomes.

## Materials and methods

The study protocol was approved by the Institutional Review Board of the Cancer Institute Hospital of the Japanese Foundation for Cancer Research (2025‑GB‑018, May 14, 2025), with a waiver of informed consent. This single-centre retrospective cohort study, involving patients who underwent MIE, was conducted in accordance with the Strengthening the Reporting of Observational Studies in Epidemiology (STROBE) guidelines [13].

Patient selection and data collection

A historical cohort study was conducted involving patients who underwent MIE, including thoracoscopic or robot-assisted esophagectomy, for esophageal cancer and/or esophagogastric junction cancer between January 2010 and December 2024. Patients receiving preoperative hemodialysis were excluded, as were those who underwent a two-stage esophagectomy, given that the two-stage approach may be less invasive than a one-stage procedure. Patients with missing data were excluded from the analysis.

Patient, surgical, anesthetic, and laboratory data were manually extracted from electronic medical records. Collected variables included demographic characteristics (age, sex, height, body weight, body mass index, American Society of Anesthesiologists physical status, and smoking history (Brinkman index)) as well as comorbidities (hypertension, asthma, chronic obstructive pulmonary disease, heart disease, peripheral vascular disease, and chronic kidney disease (CKD)). Additional information obtained comprised prior chemotherapy or radiotherapy, tumor histology, tumor location, and preoperative cancer stage. Esophageal cancer staging was determined according to the 8th edition of the Union for International Cancer Control (UICC) Tumor Node Metastasis (TNM) Classification of Malignant Tumors [14]. Preoperative medications assessed included angiotensin-converting enzyme inhibitors, angiotensin II receptor blockers, steroids, non-steroidal anti-inflammatory drugs, statins, and diuretics. Preoperative laboratory parameters, hemoglobin, platelet count, albumin, aspartate aminotransferase, alanine aminotransferase, total bilirubin, sCr, and estimated glomerular filtration rate (eGFR) were retrieved from computerized records. CKD was defined as an eGFR of less than 60 mL/min/1.73 m² at baseline. Surgical and anesthetic variables recorded included the use of thoracoscopy or robot-assisted surgery, the organ and route used for reconstruction, maintenance anesthetic technique (inhalational or total intravenous anesthesia), thoracic epidural anesthesia, duration of surgery and anesthesia, and the type and volume of intraoperative fluids administered (crystalloid, hydroxyethyl starch, or albumin). Intraoperative transfusion of red blood cells, fresh frozen plasma, or platelets, as well as urine output and estimated blood loss, were also documented. As outcome measures, the primary endpoint was prolonged postoperative hospital stay (≥ 25 days), as described below, and secondary endpoints were mortality, length of hospital stay, and need for mechanical ventilation, irrespective of whether patients were extubated in the operating room.

Surgery and anesthetic management

At our institution, the standard anesthetic approach for minimally invasive esophagectomy consisted of combined general and epidural anesthesia. Epidural patient-controlled analgesia was routinely employed for postoperative pain management; when epidural anesthesia was not feasible, intravenous patient-controlled analgesia was used as an alternative. At the conclusion of surgery, patients were typically extubated in the operating room, provided that no safety concerns-such as a heightened risk of postoperative respiratory failure-were present. Anesthetic management beyond these principles, such as the selection of maintenance anesthetic agents and analgesic medications, was left to the discretion of the attending anesthesiologist.

Definitions of AKI and subclinical AKI

Postoperative AKI was defined based on the creatinine criteria established by the Kidney Disease: Improving Global Outcomes (KDIGO) guidelines [15]. AKI was diagnosed if the postoperative sCr level increased to ≥1.5 times the preoperative baseline within 72 hours after surgery, or if it rose by ≥0.3 mg/dL within 48 hours during the 72-hour postoperative observation period. This study was retrospective, and sCr, which is routinely measured at our institution, was used as an indicator of renal function. The KDIGO urine output criteria were not applied because postoperative urine output measurements and documentation were potentially incomplete or insufficiently reliable for accurate assessment.

**Figure 1:**
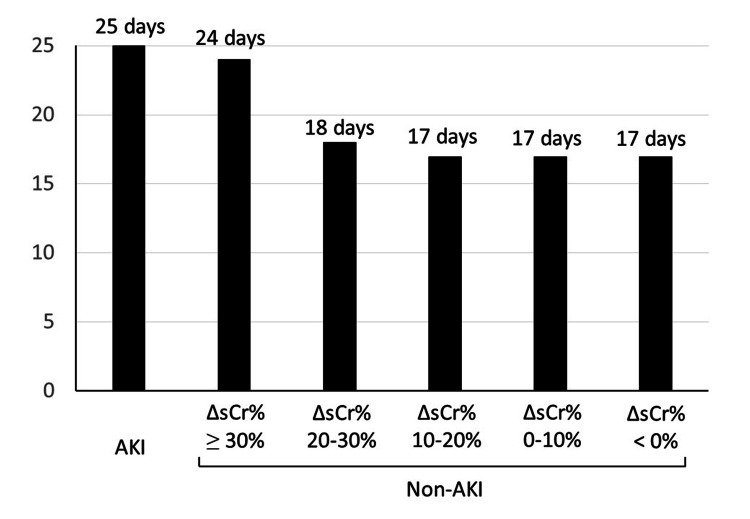
Comparison of median postoperative hospital stays between AKI and non-AKI patients (pilot study). AKI: acute kidney injury; ΔsCr%: maximum percent change in serum creatinine concentration after surgery; Non-AKI patients are divided into five groups according to their ΔsCr% values.

Because subclinical AKI refers to renal dysfunction in which the change in sCr does not meet the criteria for AKI, in this study, it was decided to define it as the maximum percentage change in sCr within 72 hours after surgery (ΔsCr%) compared with the preoperative value, in the same way as AKI [15]. The KDIGO definition of AKI is a combination of the risk, injury, failure, loss and end-stage (RIFLE) criteria [16] and the Acute Kidney Injury Network (AKIN) criteria [17]. Since both the RIFLE and AKIN criteria have been shown to be associated with a poor prognosis (mortality), their usefulness has been validated [16,17]. Therefore, the definition of subclinical AKI should be related to worsening outcomes; however, because the in-hospital mortality rate after MIE in our hospital was low, at 0.6% [10], mortality could not be used to define subclinical AKI in this study. Therefore, postoperative hospital stay was used instead of mortality as the outcome measure to define subclinical AKI. In an unpublished pilot study in which non-AKI patients were divided into five groups in 10% increments based on ΔsCr%, and the median postoperative hospital stay was examined together with that of AKI patients, the postoperative hospital stay was 17-18 days in postoperative non-AKI patients with a ΔsCr% < 30%, whereas the postoperative hospital stay was 24 days in those with a ΔsCr% ≥ 30%, which was very close to the postoperative hospital stay (25 days) in AKI patients (Figure 1). Therefore, in this study, postoperative non-AKI patients with a ΔsCr% ≥ 30% were classified as the subclinical AKI group, and other non-AKI patients were classified as the reference group.

Sample size estimation

A formal sample size calculation was not performed because all eligible patients within the accessible cohort were included in the study.

Statistical analyses

Continuous variables were summarized as median values with interquartile ranges (25th-75th percentiles) or as means with standard deviations. Categorical variables were presented as frequencies and percentages. Normally distributed continuous variables were compared using Student’s two-sample t-test, whereas non-normally distributed variables were analyzed with the Mann-Whitney U test. Categorical variables were evaluated using Fisher’s exact test. Based on findings from the pilot study, the number of patients with subclinical AKI was considered insufficient to support a stable multivariable model. Consequently, a multivariable logistic regression analysis to identify factors independently associated with subclinical AKI was not performed.

All statistical tests were two‑sided, and a p-value <0.05 was regarded as indicative of statistical significance. When comparing three groups, i.e., the AKI group, the subclinical AKI group, and the reference group, a p-value< 0.0167 was considered significant using the Bonferroni correction. EZR (Saitama Medical Centre, Jichi Medical University, Saitama, Japan), a graphical interface for R (The R Foundation for Statistical Computing, Vienna, Austria), was used for all statistical analyses [18].

## Results

This study included 984 patients who underwent MIE between January 2010 and December 2024. No patients had undergone preoperative dialysis. No patients met the exclusion criteria of missing data. A total of 933 patients were included in the final analysis after excluding 19 who underwent mediastinoscopic esophagectomy, 22 who received two-stage procedures, nine who underwent MIE in the lateral position, and one who was temporarily transferred to another hospital due to postoperative complications. AKI was diagnosed in 33 patients (3.5%) within 72 hours after surgery, of which 27 were stage 1, and 6 were stage 2. There were 25 patients with a ΔsCr% between 30% and 50%, and of these, 13 patients met the diagnostic criteria for AKI (an increase in sCr of 0.3 mg/dL or more within 48 hours); thus, 12 patients were classified as the subclinical AKI group. The remaining 888 patients were classified as the reference group. Of the 25 patients with ΔsCr% of 30-50%, 12 patients who were diagnosed with subclinical AKI had significantly lower preoperative sCr (0.64±0.08 vs. 0.96±0.32 mg/dL, P=0.0030) and significantly higher hemoglobin concentrations (13.5±1.8 vs. 12.1±1.3 g/dL, P=0.043) and eGFR (97.2±14.4 vs. 66.6±19.6 mL/min/1.73 m^2^, P=0.00021) than the 13 patients who were diagnosed with AKI.

The preoperative hemoglobin concentration was significantly higher in the subclinical AKI group than in the AKI patients. The prevalence of CKD and preoperative sCr were significantly lower in the subclinical AKI group than in the AKI group. The preoperative eGFR was significantly higher in the subclinical AKI group than in the AKI group and the reference group (Table 1).

**Table 1:** Patients’ preoperative characteristics. AKI: acute kidney injury; SD: standard deviation; BMI: Body mass index; ASA-PS: American Society of Anesthesiologists physical status; IQR: interquartile range; COPD: chronic obstructive pulmonary disease; ACE: angiotensin-converting enzyme; ARB: angiotensin II receptor blocker; NSAID: non-steroidal anti-inflammatory drug; AST: aspartate aminotransferase; ALT: alanine aminotransferase; eGFR: estimated glomerular filtration rate. * p<0.0167, Bonferroni correction, ** The Union for International Cancer Control (UICC) Tumor Node Metastasis (TNM) Classification of Malignant Tumors, 8th edition [14], was used for staging esophageal cancer.

	Reference (n=888)	Subclinical AKI (n=12)	AKI (n=33)	p-value (sub vs. ref)	p-value (AKI vs. Ref)	p-value (sub vs. AKI)
Demographic factor						
Age, y (SD)	65.2 (9.7)	63.9 (7.4)	69.7 (8.5)	0.65	0.0092*	0.043
Female, N (%)	198 (22.3)	0 (0.0)	3 (9.1)	0.079	0.085	0.55
Height, cm (SD)	165.0 (7.9)	166.1 (8.7)	164.3 (7.5)	0.62	0.64	0.50
Weight, kg (SD)	59.6 (11.2)	63.3 (15.3)	62.4 (10.6)	0.25	0.16	0.81
BMI, kg/m^2^ (SD)	21.8 (3.1)	22.9 (4.8)	23.0 (3.1)	0.23	0.026	0.91
ASA-PS, N (%)				0.57	0.0078*	0.30
1	115 (13.0)	2 (16.7)	1 (3.0)			
2	725 (81.6)	9 (75.0)	26 (78.8)			
3	48 (5.4)	1 (8.3)	6 (18.2)			
4	0 (0.0)	0 (0.0)	0 (0.0)			
5	0 (0.0)	0 (0.0)	0 (0.0)			
Brinkman index (IQR)	500 (120-800)	413 (0-649)	600 (140-870)	0.42	0.48	0.30
Chemotherapy, N (%)	502 (56.5)	9 (75.0)	16 (48.5)	0.25	0.38	0.18
Radiotherapy N (%)	48 (5.4)	2 (16.7)	1 (3.0)	0.14	1	0.17
Esophageal cancer						
Cancer histology N (%)				0.12	0.58	0.45
Squamous cell	765 (86.1)	8 (66.7)	27 (81.8)			
Adenocarcinoma	109 (12.3)	4 (33.3)	6 (18.2)			
Other	14 (1.6)	0 (0.0)	0 (0.0)			
Tumor location N (%)				0.29	0.82	0.57
Upper	151 (17.0)	4 (33.3)	7 (21.2)			
Middle	367 (41.3)	3 (25.0)	13 (39.4)			
Lower	370 (41.7)	5 (41.7)	13 (39.4)			
Preoperative cancer stage, N (%) **				0.13	0.73	0.34
0	5 (0.6)	0 (0.0)	0 (0.0)			
I	364 (41.0)	2 (16.7)	13 (39.4)			
II	220 (24.8)	2 (16.7)	6 (18.2)			
III	243 (27.4)	6 (50.0)	12 (36.4)			
IV	56 (6.3)	2 (16.7)	2 (6.1)			
Comorbidity N (%)						
Hypertension	339 (38.2)	6 (50.0)	22 (66.7)	0.55	0.0016*	0.32
Asthma	37 (4.2)	0 (0.0)	0 (0.0)	1	0.64	1
COPD	144 (16.2)	2 (16.7)	3 (9.1)	1	0.34	0.60
Heart disease	83 (9.3)	1 (8.3)	7 (21.2)	1	0.035	0.42
Peripheral vascular disease	16 (1.8)	0 (0.0)	2 (6.1)	1	0.13	1
Chronic kidney disease	108 (12.2)	0 (0.0)	13 (39.4)	0.38	0.0001*	0.0096*
Perioperative medication, N (%)						
ACE inhibitor	7 (0.8)	0 (0.0)	2 (6.1)	1	0.038	1
ARB	166 (18.7)	0 (0.0)	11 (33.3)	0.14	0.044	0.023
Steroid	9 (1.0)	0 (0.0)	0 (0.0)	1	1	1
NSAID	49 (5.5)	2 (16.7)	1 (3.0)	0.15	1	0.17
Statin	88 (9.9)	2 (16.7)	8 (24.2)	0.34	0.016*	0.71
Diuretic	26 (2.9)	0 (0.0)	3 (9.1)	1	0.081	0.55
Laboratory test						
Hemoglobin, g/dL (SD)	12.5 (1.6)	13.5 (1.8)	12.2 (1.4)	0.039	0.32	0.015*
Platelets, x 10^3^/µL (SD)	231 (68)	218 (100)	207 (90)	0.51	0.048	0.72
Albumin, g/dL (SD)	4.0 (0.4)	4.0 (0.3)	3.9 (0.3)	0.95	0.17	0.35
AST, IU/L (SD)	22.4 (10.8)	23.6 (3.7)	21.0 (7.6)	0.70	0.48	0.27
ALT, IU/L (SD)	18.7 (16.3)	20.7 (7.7)	16.4 (10.1)	0.68	0.41	0.19
Total bilirubin, mg/dL (SD)	0.49 (0.23)	0.51 (0.24)	0.50 (0.20)	0.74	0.79	0.87
Creatinine, mg/dL (SD)	0.76 (0.19)	0.64 (0.08)	0.93 (0.28)	0.041	0.0000*	0.0012*
eGFR, ml/min/1.73 m^2^ (SD)	79.0 (18.7)	97.2 (14.4)	66.3 (20.5)	0.0008*	0.0001*	0.0000*

Compared with the reference group, the subclinical AKI group had significantly longer operation times and anesthesia times, and the total amount of fluid administered was significantly greater. Blood loss was significantly higher in patients with subclinical AKI than in those in the reference group (Table 2).

**Table 2:** Perioperative variables. AKI: acute kidney injury; IQR: interquartile range; TIVA: total intravenous anesthesia; RBC: red blood cell. * p<0.0167, Bonferroni correction.

	Reference (n=888)	Subclinical AKI (n=12)	AKI (n=33)	p-value (sub vs. ref)	p-value (AKI vs. ref)	p-value (sub vs. AKI)		
Surgery								
Robot-assisted surgery, N (%)	138 (15.5)	2 (16.7)	4 (12.1)	1	0.81	0.65		
Reconstructed organ, N (%)				1	1	1		
Stomach	865 (97.4)	12 (100.0)	33 (100.0)					
Others	23 (2.6)	0 (0.0)	0 (0.0)					
Reconstructed route, N (%)				0.25	0.025	0.45		
Retrosternal	685 (77.1)	8 (66.7)	21 (63.6)					
Intrathoracic	83 (9.3)	3 (25.0)	3 (9.1)					
Posterior mediastinum	113 (12.7)	1 (8.3)	7 (21.2)					
Others	7 (0.8)	0 (0.0)	2 (6.1)					
Duration of surgery, min (IQR)	468 (411-540)	544 (487-575)	493 (431-579)	0.0063*	0.069	0.25		
Anesthesia								
Anesthetic agent N (%)				0.24	1	0.27		
Inhalational	869 (97.9)	11 (91.7)	33 (100.0)					
TIVA	19 (2.1)	1 (8.3)	0 (0.0)					
Epidural, N (%)	840 (94.6)	11 (91.7)	24 (72.7)	0.49	0.0001*	0.25		
Total intravenous fluid, mL (IQR)	3750 (3100-4450)	4695 (4213-5255)	4000 (3300-5100)	0.0071*	0.15	0.22		
Crystalloid, mL (IQR)	2850 (2308-3400)	3625 (2538-4000)	3000 (2450-3850)	0.078	0.38	0.38		
Hydroxyethyl starch, mL (IQR)	1000 (500-1000)	1100 (1000-1388)	1000 (500-1050)	0.026	0.38	0.15		
Albumin, mL (IQR)	0 (0-0)	0 (0-500)	0 (0-0)	0.027	0.33	0.28		
Estimated blood loss, mL (IQR)	90 (55-160)	275 (153-455)	150 (85-260)	0.0000*	0.0006*	0.051		
Urine volume, mL (IQR)	500 (320-750)	515 (444-798)	500 (250-750)	0.60	0.57	0.55		
RBC transfusion, N (%)	14 (1.6)	0 (0.0)	3 (9.1)	1	0.020	0.55		
Duration of anesthesia, min (IQR)	546 (488-616)	631 (602-662)	563 (513-648)	0.0011*	0.079	0.093		

The frequency of postoperative long-term hospitalization (≥ 25 days) as a primary outcome in the subclinical AKI group was not different from that in the AKI group, but was significantly higher than in the reference group. The frequency of the need for postoperative mechanical ventilation was significantly higher in the subclinical AKI group than in the reference group. Three patients in the subclinical AKI group required mechanical ventilation, but all were extubated in the operating room and reintubated after surgery. There was no significant difference in the in-hospital mortality rate between the subclinical AKI group and other groups (Table 3).

**Table 3:** Outcomes. AKI: acute kidney injury; IQR: interquartile range * p<0.0167, Bonferroni correction

	Reference (n=888)	Subclinical AKI (n=12)	AKI (n=33)	p-value (sub vs. ref)	p-value (AKI vs. ref)	p-value (sub vs. AKI)		
Primary outcome								
Postoperative hospital stay ≥ 25 days, N (%)	165 (18.6)	6 (50.0)	17 (51.5)	0.015*	0.0000*	1		
Secondary outcome								
In-hospital mortality, N (%)	4 (0.5)	0 (0.0)	1 (3.0)	1	0.17	1		
Hospital stay, days (IQR)	17 (14-22)	23.5 (16-33)	25 (16-32)	0.033	0.0002	0.98		
Mechanical ventilation, N (%)	29 (3.3)	3 (25.0)	9 (27.3)	0.0072*	0.0000*	1		

## Discussion

In the present study, the diagnostic criterion for subclinical AKI was defined as ΔsCr% ≥ 30% within 72 hours after MIE, and the incidence of subclinical AKI was 1.3%. There was no significant difference in mortality rates, but the frequencies of long-term postoperative hospitalization ≥ 25 days and the need for postoperative mechanical ventilation were significantly higher in the subclinical AKI group than in the reference group.

Although several international definitions of AKI have been established [15-17], there is no universal definition of subclinical AKI. The impact of slight increases in sCr after major surgery on outcomes has been investigated mainly after cardiac surgery [6,7], but in many studies, the definition of subclinical AKI has been defined arbitrarily and without sufficient evidence. In the present study, subclinical AKI was defined using ΔsCr%, which was determined based on the length of postoperative hospital stay, and there were significant differences between the subclinical AKI and reference groups not only in the frequency of patients who required long-term hospitalization, but also in the frequency of patients who required postoperative mechanical ventilation. Without the concept of subclinical AKI, patients would have been treated as a single group, a non-AKI group, but by defining subclinical AKI with ΔsCr%, patients who did not meet the diagnostic criteria for postoperative AKI could be divided into two groups in relation to outcomes.

It is generally accepted that poor preoperative renal function is a risk factor for postoperative AKI [19]. In fact, in the present study, preoperative eGFR was significantly lower in the AKI group than in the reference group, but preoperative eGFR was higher in the subclinical AKI group than in the reference group, which cannot be explained. Among patients with ΔsCr% of 30-50%, patients with a high baseline sCr (>1.0 mg/dl) were more likely to meet the criteria for AKI (an increase of 0.3 mg/dl or more within 48 hours), which may have resulted in many patients with a low baseline sCr being included in the subclinical AKI group. Because the subclinical AKI group had greater amounts of blood loss and fluid infusion, and longer surgical and anesthesia times than the reference group, the subclinical AKI patients in this study may not simply have had milder renal damage than AKI patients, but may have had a different pathophysiology from that of AKI.

Several hypotheses can be proposed as to the mechanism by which subclinical AKI occurs, although they cannot be proven by the present study. First, AKI may not have developed despite damage reflected by the large amount of bleeding in the subclinical AKI group because preoperative renal function was better in the subclinical AKI group. In addition, the longer surgery and anesthesia times and the larger amount of fluid administered in the subclinical AKI group than in the reference group may have resulted in greater dilution of serum creatinine, which may have led to greater underestimation of the postoperative decline in renal function. Furthermore, although there was no significant difference in the frequency of epidural anesthesia between the subclinical AKI group and the AKI group, the frequency in the subclinical AKI group was as high as that in the reference group. The renal protective effect of epidural anesthesia in the subclinical AKI group may have resulted in the degree of renal damage being mild, not meeting the diagnostic criteria for AKI [5]. The unique patient background of the subclinical AKI group is also evident from the significant differences in preoperative sCr and eGFR between patients diagnosed with AKI and with subclinical AKI, with ΔsCr% in the same range of 30-50%. The mechanisms proposed for the development of subclinical AKI remain speculative and require further verification.

The frequency of requiring postoperative mechanical ventilation was significantly higher in the subclinical AKI group than in the reference group (Table 3), which may have been a factor resulting in the prolonged postoperative hospital stay. The reasons for the high frequency of need for mechanical ventilation in the subclinical AKI group in the present study are unclear, but they may be explained, at least in part, by the interaction of kidney and lung pathologies, which is known as lung-kidney crosstalk [20]. Lung injury from renal dysfunction can result from volume overload and other mechanisms such as the release of inflammatory cytokines, endothelial apoptosis, and necroinflammation [21]. A retrospective study by Li et al. [22], examining risk factors for postoperative pulmonary complications after MIE, showed a significant association between postoperative pulmonary complications and postoperative creatinine levels, suggesting that renal dysfunction after MIE may potentially affect lung pathology.

Since postoperative AKI causes worse outcomes, and there is no effective treatment other than symptomatic treatment for AKI, prevention is considered to be the most important AKI countermeasure performed by anesthesiologists [23]. Therefore, attempts have been made to search for risk factors for AKI after MIE [5]. However, since the present study showed that renal dysfunction (ΔsCr ≥30%) that does not meet the diagnostic criteria for AKI is associated with worsening outcomes, it appears that anesthesiologists should recognize not only postoperative AKI, but also subclinical AKI as targets for prevention.

Several limitations of the present study warrant consideration. First, as with all observational studies, residual or unmeasured confounding may provide alternative explanations for the findings, and the generalizability of the results is restricted to institutions with patient populations and surgical practices comparable to those in this study. Second, although intraoperative hypotension is a well‑recognized risk factor for AKI in noncardiac surgery, detailed intraoperative hemodynamic data were not available for analysis [24]. Accordingly, we were unable to assess the frequency of hypotension during surgery. Third, we did not evaluate intraoperative drug use that could potentially influence the risk of perioperative AKI. Fourth, clear diagnostic criteria for comorbidities were not applied, which may have led to misclassification. Fifth, this study only used the KDIGO serum creatinine criteria to define AKI and did not take into account urine output. Finally, the sample size was relatively small, necessitating inclusion of the entire cohort and precluding a formal sample size calculation. Our institution is a high-volume centre that has recently performed approximately 120 cases of MIE per year, but the incidence of subclinical AKI is 1.3%, with only 1-2 cases occurring per year. Therefore, multi-institutional collaborative studies will be necessary to investigate subclinical AKI in greater detail. Due to the small number of subclinical AKI patients, it was not possible to identify risk factors for subclinical AKI through multivariate logistic regression analysis.

## Conclusions

Defining subclinical AKI as ΔsCr% ≥ 30% within 72 hours after MIE, the incidence was found to be 1.3%. Patients with subclinical AKI more frequently required mechanical ventilation after surgery than other non-AKI patients (reference group). This exploratory study may suggest that not only AKI, but also subclinical AKI in the early postoperative period should be targeted for prevention in patients undergoing MIE. Larger multicenter studies may be needed to clarify the pathophysiology and risk factors of subclinical AKI.
